# Mechanisms of suicidal behavior development in military personnel

**DOI:** 10.1192/j.eurpsy.2025.911

**Published:** 2025-08-26

**Authors:** S. Moroz, V. Semenikhina, M. Sheludenko

**Affiliations:** 1 MUNICIPAL ENTERPRISE DNIPROPETROVSK MULTIPROFILE CLINICAL HOSPITAL FOR PROVISION OF PSYCHIATRIC CARE » DNIPROPETROVSK REGIONAL COUNCIL, Dnipro, Ukraine

## Abstract

**Introduction:**

Suicide risk correlates with the nature of psychotraumatic factors that act directly during military service, their severity, duration of exposure, and individual adaptive capacities of military personnel. The development of suicidal behavior occurs against the background of anxiety and depressive syndromes in military patients and is aggravated by concomitant addictive behavior.

**Objectives:**

To investigate the relationship between the mechanisms of development of suicidal behavior in the military and psychopathological status that potentiate suicidal activity.

**Methods:**

The following diagnostic scales were used: Suicide Risk Scale of the Los Angeles Suicide Center, Sad People Scale (W.M. Patterson, H.H. Dohn, J. Bird), Spielberger-Hanin Scale of Reactive (Situational) and Personal Anxiety (STAI), Hospital Anxiety and Depression Scale (HADS), Hamilton Rating Scale for Depression, Impact of Event Scale (IES-R). Review of existing literature on suicidal behavior among military personnel.

**Results:**

38 patients with a suicide attempt (within the diagnostic criteria F 43, F 32, F 31, F07 according to ICD-10) were examined during 6 weeks of inpatient care. It was established that the psychopathological presuicidal period in the examined patients was formed against the background of high levels of anxiety (38% of patients), anhedonia (26% of patients), feelings of sadness and hopelessness (19% of patients), addictive behavior (13% of patients), feelings of anger and irritability (4% of patients). In 43% of cases, the formation of suicidal behavior in military personnel was observed against the background of violations of interpersonal relationships: self-isolation behavior (22%), conflict (13%), and antisocial actions (8%). The duration of the pre-suicidal period are several minutes (23% of patients), several days (44% of patients), a month or more (33% of patients). The dominant methods of suicide attempts among the observed military personnel are self-inflicted wounds (61%), poisoning (18%), gunshot wounds (11%), hanging attempts (8%) and self-arson (2%). The reaction of suicidal people to an unsuccessful suicide attempt depends on the current mental state of the military personnel, often with a critical assessment, less often - suicidal-fixed.

**Conclusions:**

In most cases, the development of suicidal behavior in military personnel occurs against the background of anxiety, depressive symptoms, post-concussion disorders, anhedonia, and concomitant addictions. Prevention of suicide among military personnel should include early detection of the main psychopathological symptoms and timely crisis therapy.

**Disclosure of Interest:**

None Declared

